# Therapeutic control and resistance of the EGFR-driven signaling network in glioblastoma

**DOI:** 10.1186/s12964-015-0098-6

**Published:** 2015-03-31

**Authors:** Francisco Azuaje, Katja Tiemann, Simone P Niclou

**Affiliations:** Department of Oncology, NorLux Neuro-Oncology Laboratory, Luxembourg Institute of Health (LIH), Luxembourg, Luxembourg

**Keywords:** Epidermal growth factor receptor (EGFR), Targeted therapies, Resistance to treatment, Glioblastoma, Systems biology

## Abstract

The alteration of the epidermal growth factor receptor (EGFR)-driven signaling network is a characteristic feature of glioblastomas (GBM), and its inhibition represents a treatment strategy. However, EGFR-targeted interventions have been largely ineffective. Complex perturbations in this system are likely to be central to tumor cells with high adaptive capacity and resistance to therapies. We review key concepts and mechanisms relevant to EGFR-targeted treatment resistance at a systems level. Our understanding of treatment resistance as a systems-level phenomenon is necessary to develop effective therapeutic options for GBM patients. This is allowing us to go beyond the notion of therapeutic targets as single molecular components, into strategies that can weaken cancer signaling robustness and boost inherent network-level vulnerabilities.

## Introduction

The epidermal growth factor receptor (EGFR) signaling network is crucial in the regulation of cancer cell proliferation, migration and survival. It has been significantly implicated in the initiation and progression of glioblastoma (GBM), a very aggressive class of brain cancer [1-3], where the median survival time of GBM patients is approximately 15 months [4]. GBM is very difficult to treat and always recurrent in the large part due to its highly invasive and chemo-resistant properties. In general, cancer resistance to treatment is characterized by either: a lack of tumor response to the treatment, a decreased sensitivity to the treatment, or progression of the tumor after a partial response to treatment. GBM’s sensitivity to treatment, in particular to tyrosine kinase inhibitors (TKI), and the sources of potential resistance associated with the underlying biological interaction networks deserve novel investigations with alternative approaches.

The relevance and oncogenic roles of the EGFR-driven signaling network in GBM have been extensively probed with *in vivo* and *in vitro* models, as well as with different analytical approaches. The latter have ranged from histopathological and genetic studies to large-scale “omics” research involving clinical samples. Different components of this signaling system exhibit aberrant behavior in the majority of GBM tumors [5].

In GBM the capacity of the EGFR signaling network to resist targeted therapies has been shown [6,7]. Thus, further studies about the system’s responses to the inhibition of its components are required, particularly in the context of relevant patient-derived *in vivo* models. In terms of progression-free survival, the response of GBM patients to treatment with EGFR TKIs has been largely ineffective [3,8,9]. For instance, Vivanco et al. [3] indicated that therapeutic failure may be in part explained by insufficient levels of EGFR inhibition, and that targeting its inactive conformation may be a more effective strategy. Other investigations have suggested that responsive patients tend to display the mutated EGFR variant III (EGFRvIII), or amplified EGFR, together with preserved PTEN function [10,11]. However, this has not been consistently and independently verified in clinical trials. In addition, the mechanisms through which the EGFR-driven signaling network contributes to adaptation and treatment resistance deserve wider characterizations beyond the traditional “linear pathway” view of signaling, into one of integrative interaction networks.

These observations underscore: a. the complexity of the EGFR-driven signaling network in GBM, and b. our relatively limited understanding of its dynamic properties at the systems level. The discovery of potentially effective treatments that target the EGFR-driven signaling network will rely on our ability to identify systems-level mechanisms underlying its resistance to therapy. This also entails a better understanding of the interplay between specific molecular perturbations, such as genomic aberrations, and systems-level emergent behaviors.

The remainder of this review begins with an introduction to the EGFR-driven signaling network and to key aberrations observed in GBM. We then frame the problem of treatment resistance as a consequence of intrinsic systems-level robustness. We synthesize fundamental mechanisms that can contribute to the acquisition of resistance against perturbations. Specifically, we discuss: diversity and redundancy, modularity, feedback controls and spatio-temporal dynamics. A characterization of these properties will deepen our understanding of how tumor cells can adapt to therapeutic interventions. We conclude this review with perspectives on implications and challenges for new therapeutic research.

### Overview of the EGFR-driven signaling network and major aberrations

The receptor tyrosine kinase (RTK) EGFR is one of the four members of the ErbB family. It consists of an extracellular ligand binding region, which is connected to the cytosolic region through a hydrophobic transmembrane domain. The main ligand of EGFR is the epidermal growth factor (EGF), but it can also be regulated by other six known ligands: TGF-α, amphiregulin, epigen, heparin-binding EGF-like growth factor (HB-EGF), epiregulin and betacellulin [12].

**Figure 1:**
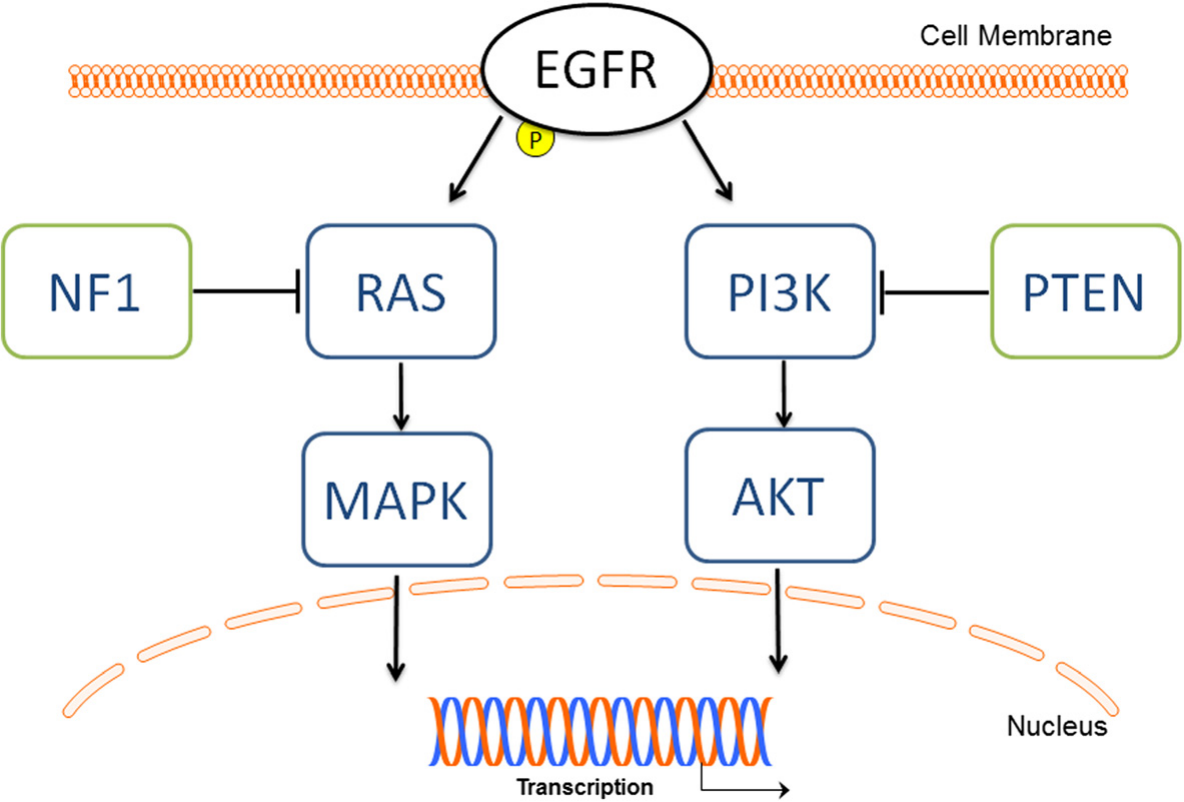
**Schematics of EGFR signaling via PI3K and AKT.** Graphics depicting cell membrane, nucleus and transcription taken from motifolio.com. This is an oversimplified view of the network. In reality, for example, crosstalks between different canonical pathways, such as between RAS and PI3K, and multiple feedback loops are also observed as discussed in the next sections.

Ligand binding results in an active dimeric conformation of EGFR; by either forming a complex with another EGFR (homodimerisation) or with one of the other ErbB family members (heterodimerisation) [13,14]. Upon dimerization, the catalytic intracellular domain is activated by phosphorylation of tyrosine residues and results in the recruitment of different cytosolic adapter proteins. Proteins containing a Src homology domain 2 (SH2) region recognize tyrosine phosphate residues and bind directly to the activated receptor. Such proteins become activated and transfer the signal to downstream effectors [15,16]. EGFR can activate different signal transduction pathways in parallel; the most prominent ones are the RAS/MAPK and the phosphatidylinositide 3 kinase (PI3K)-AKT pathways (Figure 1).

Growth-factor-receptor bound-2 (GRB2) is a SH2-domain containing protein, which forms a receptor-bound complex with a guanine-nucleotide exchange factor (GEF) called SOS (Son of Sevenless). Such complexed SOS activates the G-protein Ras by exchanging guanosine diphosphate (GDP) for guanosine triphosphate (GTP) [16,17]. The activated Ras triggers a downstream signaling cascade with mitogen activated protein kinases (MAPKs), which can phosphorylate a nuclear protein called Jun. Jun forms complexes with other nuclear proteins to form the transcription factor activator protein 1 (AP-1). The latter is a key transcription factor, which causes transcription and translation of proteins responsible for cell growth and division. Activated Ras is shut down by GTPase activating proteins (GAPs), which exchange GTP to GDP to avoid permanent signaling. One such Ras-GAP is the tumor suppressor neurofibromin 1 (NF1), the only GAP that is shown to act in tumorigenesis [18,19].

PI3K, another important downstream effector of activated EGFR, is a SH2-containing signal transducer enzyme, which regulates apoptosis and hence cell survival through its downstream effector AKT. Upon EGFR activation and phosphorylation, PI3K is recruited to the cell membrane, where it phosphorylates phosphatidylinositol 4,5-bisphosphate (PIP2) to phosphatidylinositol (3,4,5)-trisphosphate (PIP3). AKT can interact with PIP3, followed by a phosphorylation of AKT at Threonin308 by phosphoinosite-dependent protein kinase-1 (PDK1). To develop its full activity, AKT is phosphorylated at a second amino acid, Serine 473, by the mammalian target of rapamycin complex 2 (mTORC2). Activated AKT can transduce the signal by phosphorylation of several downstream substrates, such as the transcription factor FOXO, resulting in the regulation of numerous processes such as proliferation and cell survival. The tumor suppressor phosphatase and tensin homolog (PTEN) is an important negative regulator of the PI3K/AKT pathway. It dephosphorylates and subsequently delocalizes PIP3 from the cell membrane: This results in a relocalization of AKT to the cytoplasm, where it cannot be activated anymore. Beside alterations in RAS and PI3K, mutations or deletions of negative regulators, e.g., NF1 or PTEN, are very common in cancer. As a result EGFR signaling is not terminated allowing cells to proliferate and survive without restraint [5,20,21] (Figure. 1).

Altered EGFR signaling has been implicated in the emergence and progression of cancers by dysregulated and uncontrolled cell growth [22,23]. It has been shown that in 90% of GBMs RTK signaling is dysregulated, either by aberrant expression or mutations of the receptors themselves or of their downstream effectors. The most frequently amplified or mutated RTK network in primary GBM is EGFR. Amplification and overexpression of EGFR is found in more than 50% of GBMs [24,25]. The overexpression of the receptor may also be accompanied by an increased expression of its activating ligands [22], which suggests the existence of a positive feedback loop that reinforces the aberrant signaling.

In GBM, EGFR gene amplification often is accompanied by an additional gene rearrangement, of which the truncated EGFR variant III (EGFRvIII) is the best characterized alteration in GBM. Therefore, these tumors coexpress wild type EGFR (EGFRwt) and the mutant EGFRvIII [1,24,26]. The truncation of EGFRvIII results from an in-frame deletion of the coding sequence from exon 2-7. This leads to a loss of the extracellular domain, thus directly affecting its ligand binding potential [27,28]. Despite its inability to bind ligands, EGFRvIII is reported to be constitutively active [21,25,29]. One reason for this constitutive activation is the reduced interaction with the E3-ligase Cbl, which in turn results in a decreased ubiquitin-induced receptor degradation [30]. This represents an evasion mechanism from a negative feedback control, which is critical for terminating signaling and diminishing tumorigenic activity. Aberrations in the EGFR-driven signaling network induce hypo- or hyper-activation of multiple downstream pathways. This can be the result of different effectors and include multiple dysregulated feedback control mechanisms.

### Treatment resistance as a phenomenon of intrinsic system robustness

In GBM, the targeting of the EGFR pathway has been investigated with different approaches, including receptor kinase inhibitors, such as erlotinib and gefitinib, and antibodies, such as cetuximab. These approaches have often shown promising results in cell-based assays and preclinical models. However, they have been largely disappointing in patient treatment [31].

EGFR kinase inhibitors such as geftinib, erlotinib and lapatinib have been approved to treat lung and breast cancers [11,32], but failed in GBM tumors. Although these disappointing results may be partially explained by the fact that the drugs cannot reach their target cells in the brain because of the blood brain barrier [33,34]. There is also evidence that TKIs can indeed enter human brain tumors in elevated concentrations and dephosphorylate EGFR [35]. In this situation, treatment resistance may be understood as a manifestation of the systems-level robustness of cancer cells to external stimuli [36,37]. The robustness of a system can be defined as its ability to preserve a particular state in the presence of perturbations. This entails the maintenance of function (dysfunction) or biological stability, and is believed to represent an effective evolution mechanism. Systems-based properties, such as feedback loops and functional redundancy in the structure of biological networks, have been shown to be the underlying cause for the sustained or recurrent molecular behavior of pathways in different organisms. Extensive network rewiring, including feedback control mechanisms, can be activated in response to drugs to maintain proliferation responses and survival [38-40] (Section on systems-level properties). In the context here discussed, a tumor cell is deemed resistant when it “shortcuts”, “compensates for” or “bypasses” receptor inhibition by establishing robust activation of survival pathways downstream of the EGFR or through other RTK signaling activation. One can also postulate that the biological properties that confer robustness to systems in their “normal” state also underlie the resistance capacity in the pathological states [37,41]. The EGFR-driven signaling network, as a key player of cell proliferation and differentiation, is one of the many systems exploited by cancer cells to enable their survival in the presence of environmental perturbations. The systems-level features discussed in the next sections are also observed in other cancer types and other signaling networks. Although our focus is on GBM and the EGFR-driven signaling network, when appropriate we also draw examples from different oncology areas to expand GBM-related discussions.

Different systems-level features underpinning such a resistance capacity are encoded in the EGFR-driven signaling network. The remainder of this article focuses on such regulatory and signaling control properties. Here we discuss four fundamental properties and provide examples of clinical interest: Component diversity and redundancy, modularity, feedback control loops, and spatio-temporal dynamics.

### Systems-level properties of EGFR signaling contributing to resistance

#### Diversity and redundancy

The EGFR-driven signaling network can maintain its function by taking advantage of proteins that are encoded by different genes, but which are capable to perform the same or similar molecular tasks. This diversity of network components and pathways then represents an important source of functional redundancy, which can act as a “fail-safe” mechanism in cancer signaling [37]. This means that when a protein or pathway is inhibited new components can be activated to preserve the anomalous molecular state. Thus, resistance may be acquired through the existence of heterogeneous network components with redundant functionality. In cancer, diversity can be enhanced through the generation of variation at the genomic and epigenomic levels. For example, genetic alterations involving point mutations and other structural sequence alterations represent an important source for the generation of functional redundancy, which can contribute to resistance to treatment [31].

**Figure 2:**
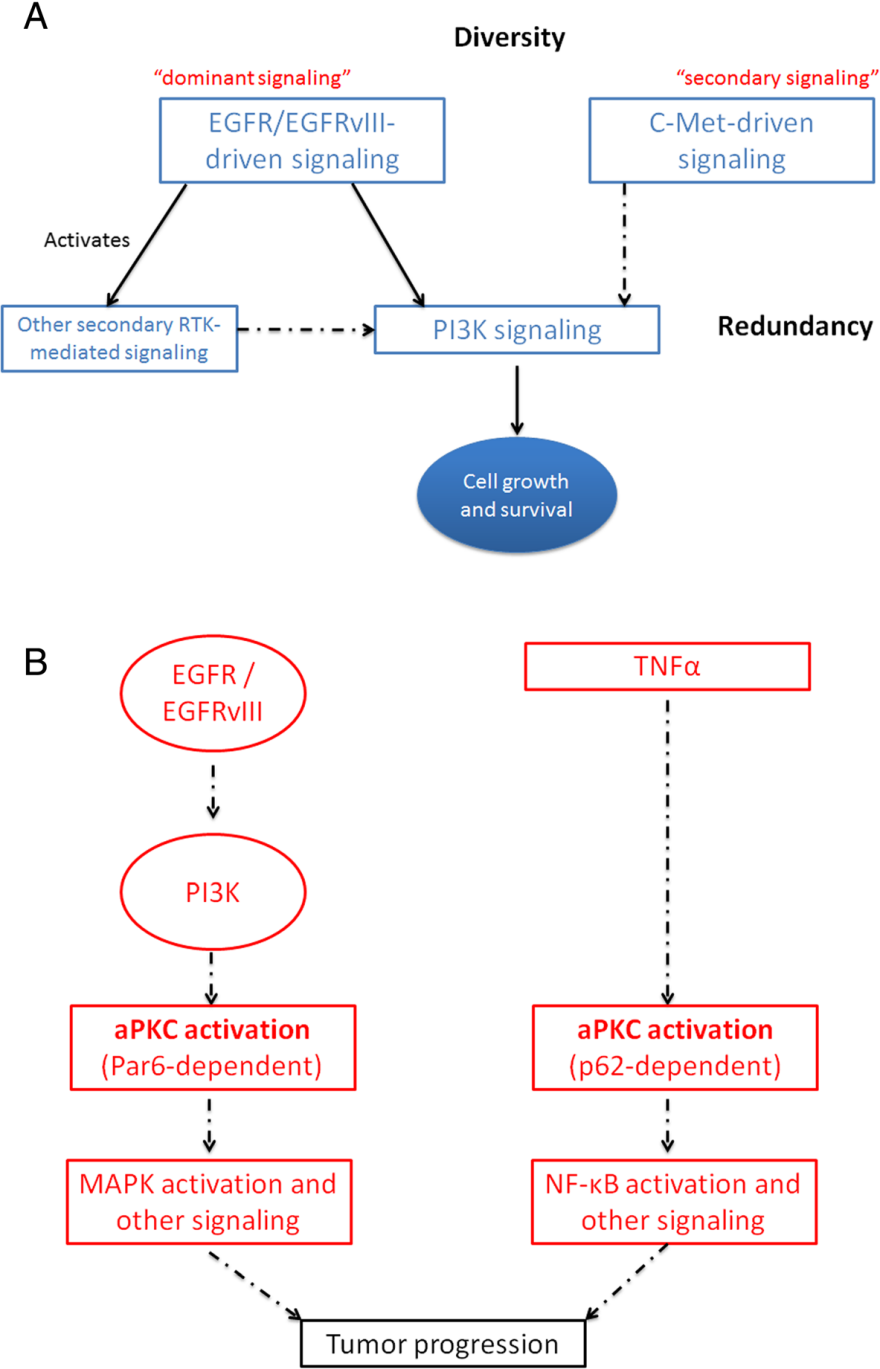
**Examples of functional diversity and redundancy involving the EGFR-driven signaling system. A.** Cross-communication between “dominant” and secondary” RTK signaling pathways and their alternating activation. Details are offered in [47]. **B.** An example of alternate signaling pathways downstream of EGFR and leading to glioblastoma progression. In both pathways a central protein (aPKC) is the key signaling component, but the resulting complexes are formed in a different fashion and alternative oncogenic signals are relayed, which eventually converge to a common outcome: tumor progression (diagram adapted from Kusne et al., [51]).

The cross-talk between the EGFR-driven signaling network and other RTK pathways is an archetypal example of diversity and redundancy, which can enhance system robustness and resistance. In GBM, the interplay between alternative RTK pathways can be exploited by tumor cells to enable the activation of oncogenes in the face of EGFR inhibition [42]. This has been observed, for example, in the case of the co-activation of c-MET (Met proto-oncogene) and EGFR-induced signaling. When treated with EGFR TKIs, c-Met keeps activation of PI3K signaling and cell survival via the recruitment of the GAB1-PI3K complex, which is normally associated with EGFR [43]. This suggests that the switching of oncogenic signaling is based on the existence of “dominant” and “secondary” RTK pathways [42]. In the previous example, EGFR and c-Met represent the dominant and secondary signaling components respectively. Hence, in this scenario resistance is a by-product of the re-wiring of the signaling web when the dominant pathway is perturbed (Figure 2A). Efforts to overcome this robustness mechanism has involved, for instance, the application of foretinib, which is capable to inhibit different RTKs: Axl, c-MET and VEGFR [44,45]. This treatment has shown promising results in mouse models: tumor reduction of lung metastasis [44], and a decline of lapatinib-triggered resistance in breast cancers [45].

Tumorigenesis and treatment resistance in GBM can be mediated by different downstream signaling pathways that may lead to the same outcome. For instance, EGFRvIII-driven growth and invasion have been associated with the independent activation of PI3K/AKT, SRC family kinases (SFK) and MAPK signaling cascades [46]. This diverse-yet-redundant signaling functionality provides the tumor cell with another robust mechanism to adapt to treatment and preserve survival advantage (Figure 2B).

Cancer cells can exploit diverse interrelated signaling components to amplify oncogenic EGFR-driven network activity. In glioblastomas, the membrane receptor DCBLD2 has recently shown to be a key mediator of the enhancement of (EGFR-stimulated) AKT signaling activity [47]. Feng et al. [47] demonstrated that: a. DCBLD2 is highly expressed in patient-derived glioblastoma samples, b. its interaction with TRAF6 is facilitated by EGFR/EGFRvIII, and c. such an interaction is used to boost AKT signaling and tumorigenesis.

The Rho family, a member of the Ras superfamily, comprises dozens of known proteins, such as Rho, Rac and Cdc42 [48]. In glioblastomas, inhibition of EGFR has been shown to modulate Rho signaling and reduce cell motility [49]. Rho proteins share multiple overlapping activators, inhibitors and downstream effectors, and some members of this family have been shown to be relevant mediators in glioblastoma invasion [50]. For example, Cdc42 and RhogG share a set of partially overlapping activators and downstream effectors with Rac1, which is activated “downstream” of Cdc42 and RhogG in the route to cell migration [48].

Kusne et al. [51] has recently reported a novel example of the activation of parallel, redundant signaling pathways downstream of EGFR, which lead to glioblastoma progression. In their investigation a central protein, aPKC, is responsible for triggering two alternative pathways that individually are sufficient to drive tumor progression (Figure 2B). One of the pathways involves the EGFR-driven activation of PI3K, aPKC, MAPK and other oncogenic signals. The second one is EGFR-independent, initiated by TNFα, also mediated via aPKC complex formation, and requires downstream activation of NF-κB. The key distinguishing feature of these glioblastoma progression routes is the use of different adaptor proteins for aPKC activation: Par6 and p62 for EGFR- and TNFα-directed signaling respectively [51]. Thus, the resulting complexes are formed in a different fashion and alternative oncogenic signals are relayed, which eventually converge to a common outcome: tumor progression (diagram adapted from [51]).

Despite these advances, further investigations will be needed to conclusively demonstrate the mechanisms by which systems diversity and redundancy can enable resistance to treatment.

### Modularity

A module can be defined as a set of highly interconnected, functionally-related components in a biological interaction network. Protein complexes, co-regulated genes and other specialized signaling pathways are examples of modularity. Control feedback circuits (Section “Feedback control mechanisms”) can also be regarded as modules. Modularity is not only a property inherent to network connectedness, but also encoded in temporal and spatial boundaries of molecular activity. The organization of signaling networks on the basis of such physical or functional building blocks allows these systems to deploy additional adaptation and resistance mechanisms. This is because modularity allows cells to separate component- or module-specific alterations from the overall activity of the system. This effectively offers multiple “decoupling” or “buffering” mechanisms that contribute to the capacity of the system to resist perturbations, including external environmental stimuli [37].

**Figure 3:**
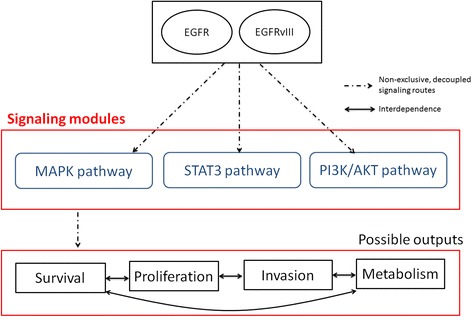
**Examples of modularity in the EGFR-driven signaling system.**

The PI3K, MAPK and STAT3 signaling pathways are examples of modules downstream the EGFR- and EGFRvIII-driven signaling networks (Figure 3). The MAPK module, which specializes in cell proliferation, includes the activation of multiple proteins such as GRB2, RAS, RAF, MEK, ERK1 and ERK2. Similarly, the PI3K module offers an alternative route to cell proliferation that comprises the interplay of GAB1, PI3K, AKT and mTOR. Tumor cells may exhibit a preferential activation of specific signaling modules to resist treatment. In GBM, the preferential activation of the PI3K pathway, instead of either the STAT3- or MAPK-specific cell proliferation signaling modules, has been reported in EGFRvIII-expressing cells [52].

System modularity also offers a robust capability for the dynamic decoupling of signaling pathways. Tumor cells can exploit this to rewire cancer-related networks in the presence of treatment. In gliomas, it has been shown that, in response to EGFR inhibitors, upstream and downstream signaling pathways can be linked through the removal (or bypassing) of intermediate modules. For example, an AKT-independent module controlled by protein kinase C (PKC) can be activated to trigger a direct signaling route to the PI3K and mTOR pathways [10]. In this setting, the AKT-dependent signaling module is first deactivated by treatment, and subsequently rendered dispensable through the creation of an alternative signaling track involving an AKT-independent module downstream of EGFR. Thus, in response to treatment, the dynamic interconnection of signaling modules activated an alternative adaptive mechanism for linking growth factor expression to uncontrolled cell proliferation.

Simeone et al. [53] recently reported an example of protein network modularity in glioblastoma, which can be linked to EGFR and other signaling pathways. Proteomics and computational network analysis identified a candidate “control module”, whose activity appears to be coordinated by four network hubs, including HNF4a and c-Myc. This module is shown to be dysregulated in glioblastoma samples, and in “cross-talk” with EGFR and p53 signaling pathways [53]. Although further investigations are needed to validate the relevance of these findings, this work illustrates how network modularity can be discovered through data-driven approaches, as well as the potential of these modules for diagnostic and therapeutic applications.

### Feedback control mechanisms

The existence of multiple feedback loops in the EGFR-driven signaling network is an important feature of its robustness. In general, the activation of feedback loops is crucial for cells to adapt to perturbations. Feedback loops may overlap multiple canonical signaling systems and modules. This allows them to operate at multiple levels of biological regulation or in a cross-directional manner. Defects in the implementation of feedback loops in the EGFR-driven signaling network can lead to the emergence or progression of cancers [54].

**Figure 4:**
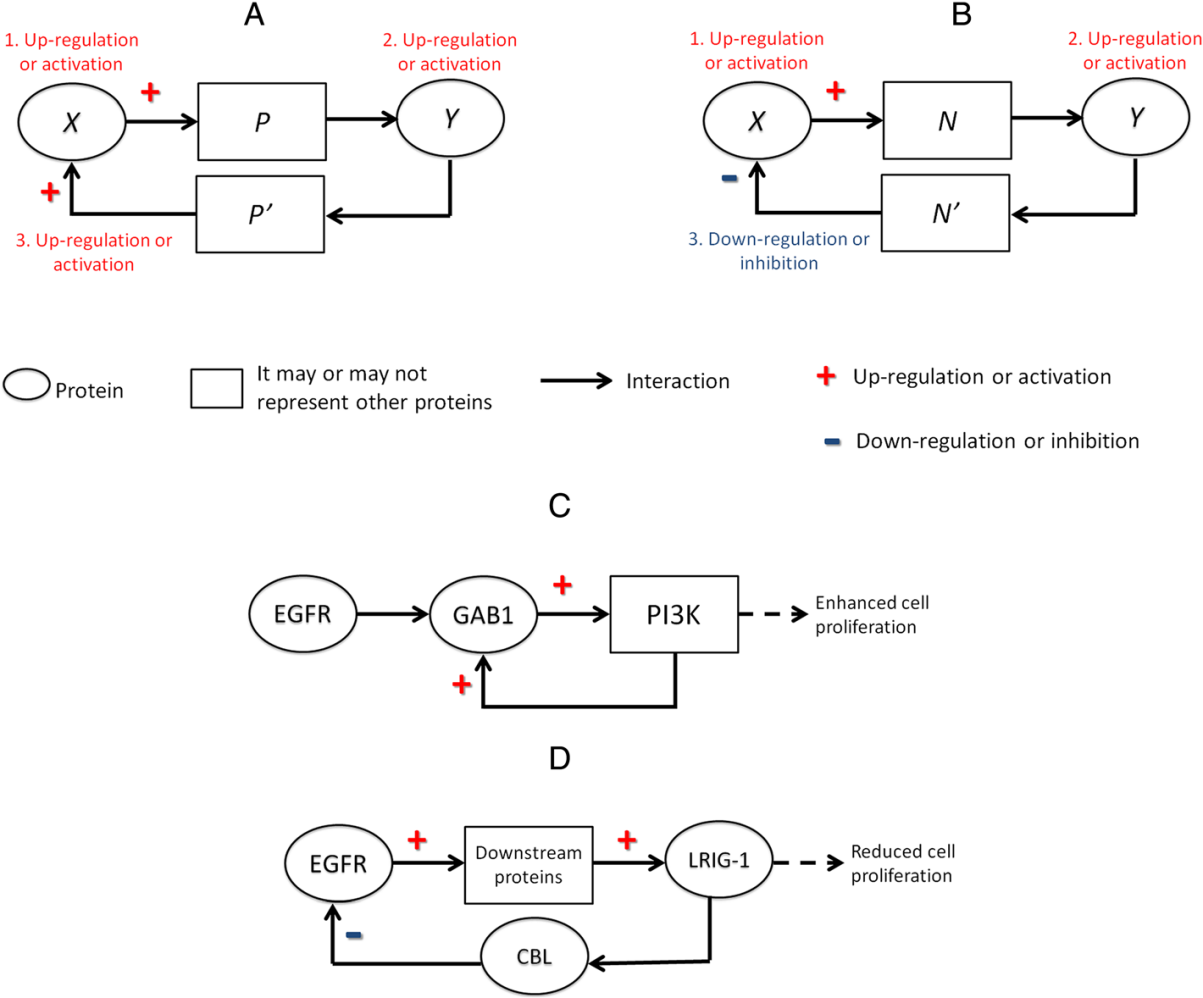
**Negative and positive feedback controls in EGFR-driven signaling system.** Schematic representations of positive **(A)** and negative feedback loops **(B)**. Example of positive feedback model **(C)**, as reported in [18], and of negative feedback in wild-type EGFR **(D)**, as reported in [55]. In **A** and **B**: numbers indicate the sequence of events.

Positive feedback is typically required to amplify input signals, and thus may be seen as a signal sensitivity-enhancing mechanism (Figure 4A). It also can be exploited to create transitions between downstream biological states, including those resembling “on-and-off switching” responses. In the EGFR and other signaling networks, signal amplification becomes a powerful mechanism to maintain the molecular state of the network when stimulated, either transiently or permanently. Conversely, negative feedback can be used to diminish input fluctuations and noise (Figure 4B). Negative feedback represents a potent mechanism to maintain stable responses in the face of diverse and stochastically variable signaling inputs. When combined, positive and negative feedbacks can also induce unstable or oscillatory responses [55].

Positive and negative feedback loops can operate at different levels in the EGFR-driven signaling process. An example of positive feedback can be described around GAB1 (growth factor receptor bound protein 2-associated protein 1). Following the activation of EGFR, GAB1 is recruited to interact with the phosphorylated sites of the receptor via the intervention of GRB2 [12]. After GAB1 undergoes phosphorylation, PI3K products containing SH2 domains are recruited to bind its PH domains. The resulting activation of PI3K molecules fosters the translocation of GAB1 to the cell membrane for further activation and interaction with PI3K molecules. In this way EGFR can indirectly activate PI3K and subsequently trigger sustained anti-apoptotic response (Figure 4C). Another example of positive feedback is the production of EGF after the EGFR-induced activation of the RAS-MAPK pathway [56].

A typical scenario of negative feedback is represented by the different dephosphorylation loops of the activated EGFR. For instance, after its activation, EGFR can recruit SHP1 and SHP2, which are SH2-domain-containing phosphatases. These proteins thus close a negative feedback loop by inducing the dephosphorylation of EGFR [12]. In GBM cell lines, it has been shown that an increase of the expression of EGFRvIII can correspond with higher levels of EGFRvIII phosphorylation [52]. However, this tendency reaches saturation after surpassing an expression threshold of approximately about 2 million copies/cell. In addition, this occurs while the phosphorylation levels of downstream proteins can continue to increase. The latter includes the activation of phosphatases that are likely to be responsible for sinking EGFRvIII phosphorylation. This represents an instance of negative feedback control of the EGFRvIII-driven signaling network that is sensitive to receptor concentration.

The activation of negative regulators of signaling can also comprise relatively delayed negative feedback loops at the transcriptional level. For instance, EGFR-driven signaling can induce the expression of multiple transcriptional repressors, such as ID2 and NAB2, which in turn can reduce the gene expression of EGF [57]. Another example centers on LRIG-1 (leucine-rich repeats and immunoglobulin-like domains 1) (Figure 4D). The activation of EGFR is typically followed by the up-regulation of LRIG-1, which in turn has been implicated in the inhibition of EGFR through the ubiquitination and degradation of EGFR. The latter is mediated through the recruitment of E3 ubiquitin-protein ligase CBL [58,59]. In GBM samples expressing EGFRvIII, however, such a CBL-mediated mechanism appears to be either unsettled or unnecessary [60]. We recently showed that soluble LRIG-1 inhibits cell growth in experimental glioblastoma models irrespective of the EGFR type or expression level [61].

The activation of specific feedback control mechanisms may not entirely depend on specific cell genotypes or perturbations. Indeed, it has been reported that their activation can be specific to cell lineage as well. This is exemplified by the differential activation of EGFR-driven feedback control in BRAF-mutant colorectal cancer and melanomas in response to BRAF inhibition [62]. Melanoma cells, unlike colorectal cancer cells, are sensitive to BRAF inhibitors. In BRAF-mutant colorectal cancer cells, BRAF keeps EGFR inactive. Hence, when BRAF is targeted this control loop is removed and pro-survival signals can be freely relayed. This does not happen in melanoma cells because EGFR is not sufficiently expressed to allow the cell to take advantage of the elimination of this feedback control [62].

Feedback circuits involving non-coding RNAs have also been investigated in glioblastomas. For instance, using glioma cells with EGFR amplification, Suh et al. [63] identified a feedback regulatory loop consisting of two miRNAs (miR-25 and miR-32), their transcriptional factors (E2F1 and MYC) and p53. In glioblastomas, these miRNAs are repressed by p53 through the inhibition of E2F1 and MYC. But on another level, the overexpression of the miRNAs results in tumor suppression *in vivo*. The latter occurs because miR-25 and miR-32 can directly target two inhibitors of p53 and the mTOR pathway: Mdm2 and TSC1. This is turn leads to the accumulation of p53 and the inhibition of cell proliferation [63]. Additional examples of regulatory circuits involving non-coding RNAs are provided below.

Although here we have put emphasis on feedback circuits, feedforward regulatory loops can also contribute to robust altered activity of EGFR-dependent signaling in glioblastoma. For example, Li et al. [64] determined a feedforward loop comprising the sequential triggering of EGFRwt, EGFRvIII and HB-EGF (a ligand of EGFRwt), which is sufficient to preserve the persistent activation of EGFRvIII signaling. In this context, overexpression of HB-EGF results in an increase of the phosphorylation of EGFRvIII. Conversely, inhibition of HB-EGF causes reduction of EGFRvIII phosphorylation [64].

### Spatio-temporal dynamics

As exemplified above with LRIG-1, a fundamental mechanism for the down-regulation of EGFR is the endocytosis of the activated receptor followed by its intracellular degradation [65]. Although it has traditionally been assumed that EGFR activation is only achieved at the cell surface, other evidence proposes that activated EGFR can initiate signaling within intracellular compartments after its internalization [66]. Moreover, it has been shown that the activity of proteins associated with such signaling varies according to specific intracellular regions. This indicates that EGFR-driven signaling is also dependent on sub-cellular localization.

**Figure 5:**
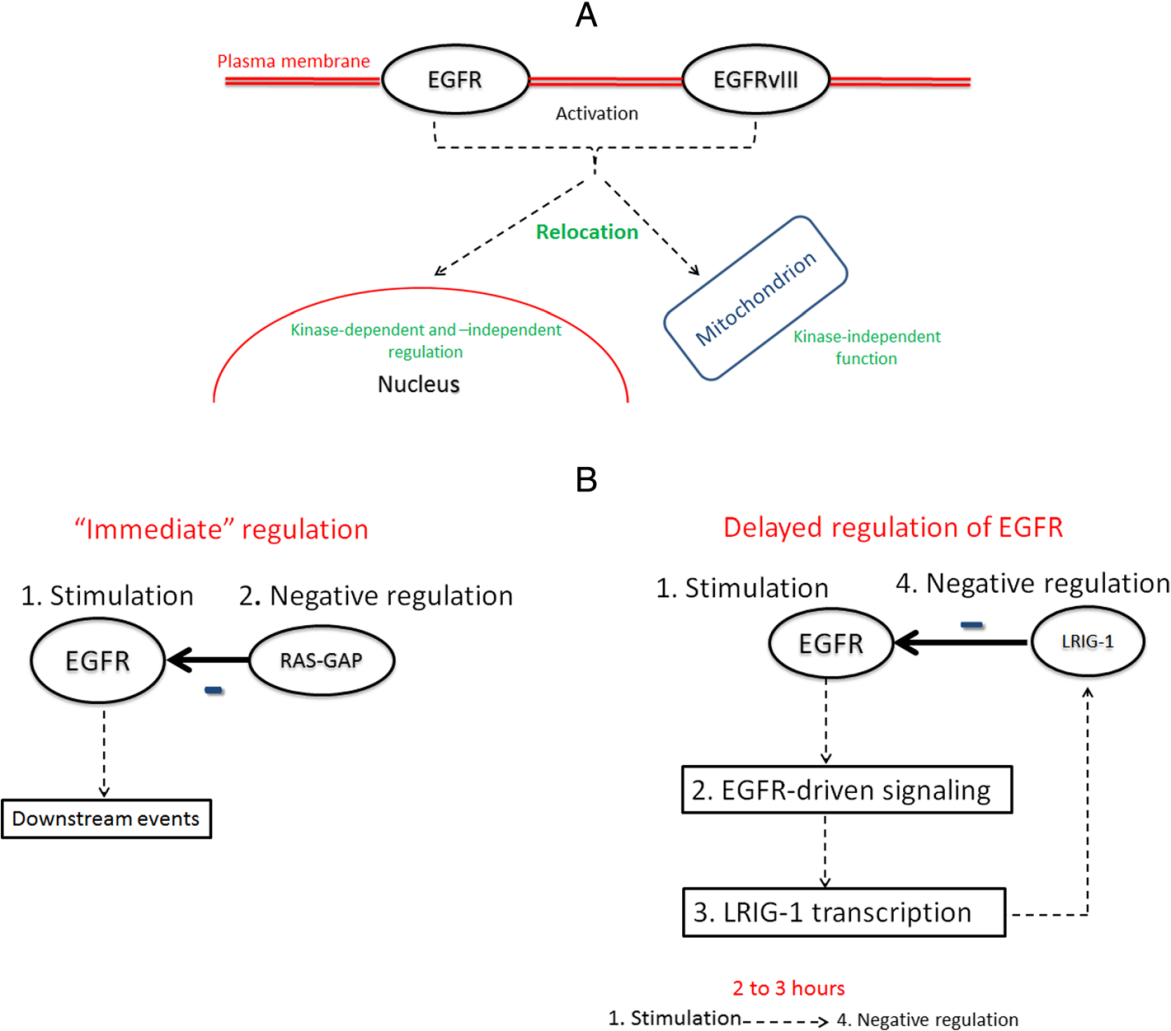
**Diversity of spatio-temporal dynamics in the EGFR-driven signaling system. A**. Example of EGFR signaling activity according to subcellular location [62]. **B**. Examples of immediate and delayed regulation of EGFR [55,69]. In **B**. numbers indicate the sequence of events.

The failure of EGFR-targeted treatments may be explained by the multiple regulatory influences that EGFR and its mutated versions can exert beyond its “classical” localization on the plasma membrane. In GBM, EGFR and EGFRvIII have been shown to be relocated to the nucleus and mitochondria in response to ligand binding and treatment [67]. This means that, in principle, signals that induce cancer growth and invasion can be relayed from different cellular localizations (Figure 5A). This can enable the tumor cell to overcome the effect of EGFR-targeted drugs that aim to disrupt kinase activity of the membrane-bound receptor.

In the nucleus, EGFR and EGFRvIII can act either as transcription factors [68] or as tyrosine kinases, and have been implicated in the transcriptional activation of oncogenes [69]. Their mitochondrial activity has been observed in response to TKI treatment and has been associated with anti-apoptotic responses [70]. There is also evidence that under hypoxia cancer cells can sustain EGFR-mediated signaling through reduced EGFR endocytosis and the preservation of the receptor in the endosome [71]. More recently, it has been shown that in a subset of glioblastomas the EGFR-driven activation of STAT3 requires: a. the phosphorylation of EGFRvIII by EFGR, b. the nuclear entrance of EGFRvIII, and b. the formation of an EGFRvIII-STAT3 complex in the nucleus [72]. Although the origin and dynamics of these translocations still merit more detailed characterizations, the subcellular localization of EGFR and its variants in response to treatment may represent a crucial factor to understanding and disabling resistance mechanisms.

The dynamics of signaling activation downstream of EGFR, as a function of time, can also provide cancer cells with potential resistance mechanisms against EGFR-targeted inhibition [54,55]. EGFR-driven signaling can occur through multiple modalities: single bursts, discrete successive bursts and multiple signaling cycles, which can be either restricted to a single cell or propagated to neighboring cells [59]. Moreover, cell proliferation can be maintained through the transient modulation of downstream signaling. For example, the continued proliferation of neuronal precursors can be accomplished through the (EGFR-induced) transient activation of ERK1/ERK2 via RAF [73]. Therefore, because a transient activation of ERK1/ERK2 is sufficient to trigger persistent proliferation, the inhibition of EGFR or other intermediate signaling components may not necessarily lead to the intended anti-proliferative effect. Actually, downstream (pro-survival) responses are not only determined by the identity of the signaling component being activated (or inhibited), but also by the strength and duration of the perturbation [74].

Negative regulators of EGFR-driven signaling, including negative feedback loops, can be activated according to different time scales [59]. A coarse classification of such regulators divides them into: a. those that require more time (from 30 minutes to hours) to be induced after receptor stimulation, and b. those that have less delayed functional activation [54,75]. The category of faster, or immediate, regulators comprise signaling components, such as CBL and RAS-GAP, that do not necessarily require *de novo* protein synthesis to perform their signaling or regulatory roles (Figure 5B). Conversely, regulators with slower response, such as LRIG-1, may first undergo transcriptional induction after receptor activation [58]. The latter is not a necessary condition though, as LRIG-1 may be already expressed [61]. This diversity of time scales and conditions for executing functional activation corroborates that EGFR-driven signaling can be adapted for the benefit, or detriment, of the cancerous cell in the presence of the same or different external stimuli. Furthermore, time-dependent differences in regulation and signaling, regardless of time scales and tumor type, are highly specific to cellular context.

Recent research also provides evidence of the circadian regulation of EGFR signaling [76]. Although these findings were obtained in breast cancer, they bring novel systems-level insights into tumor resistance and potential therapeutic approaches. Lauriola et al. [76] expand our understanding of the temporal dynamics underlying the interplay between positive and negative feedbacks required to effectively inhibit EGFR signaling. They showed that glucocorticoids can effectively inhibit EGFR-driven signaling in mice through the inactivation of EGFR’s positive feedback loops, together with the activation of negative feedbacks that are required for limiting EGFR signaling. In mice, this response is more effective during nocturnal time (active phase of the animal) when glucocorticoids are high.

### Implications for novel therapy research and further perspectives

A central question arising from this review is: Which strategies can be applied to diminish treatment resistance in the EGFR-driven signaling network? In principle, any strategy that can reduce the oncogenic robustness enabled by the systems-level properties reviewed above may represent a feasible tactic to fight treatment resistance. In practice, such approaches when applied independently may not be sufficient to kill cancer cells and, perhaps more important, to prevent further cancer cell adaptations [31]. The latter is particularly critical because, for instance, approaches to reducing system diversity may actually favor the generation of additional variation of signaling components. As further discussed below, this may include genetic aberrations or the re-wiring of existing signaling interactions. Even those strategies that aim to overcome resistance, such as combined treatments, may have the unintended effect of facilitating the accumulation of new signaling alterations that may be beneficial to tumor survival.

Effective strategies to tackle drug resistance will certainly require more comprehensive analyses and predictive models of signaling dynamics. Resistance to treatment is, inherently, a systems property that cannot be addressed by studying ligands, receptors and other signaling components in isolation. Systems-level interpretations are needed to expand the arsenal of therapeutic approaches. This may involve, for example, the inhibition of feedback loops rather than the simple removal of individual components included in the loops. Or alternatively, new feedback loops may be inserted into the signaling system to induce relevant counter-acting states (e.g., disruption of signal relays and cell dormancy) or to improve its sensitivity to serial treatments.

Perturbations or mutations that favor treatment resistance tend to be complemented by the boosting of positive feedback or the diminishing of negative feedback mechanisms [54]. These disruptions can extend the activation of EGFR-driven signaling or dampen the effect of therapeutic interventions. An example within the category of enhanced positive feedback control is the amplified activation of pathways that induce the production of EGFR ligands, following the activation of the receptor. An example of reduced negative feedback control that strengthens resistance potential is the down-regulation of suppressors of EGFR signaling, such as LRIG or CBL proteins. The elimination or weakening of the activity of these repressors are observed in tumors, including gliomas, and can shore up resistance against the targeting of the EGFR-driven signaling network. Although interventions that either enhance negative feedback or deteriorate positive feedback control loops could represent feasible strategies to combat resistance, such strategies are likely to be insufficient if applied in isolation.

The reduction of treatment resistance can also be viewed as the challenge of finding system vulnerabilities in the disease state [37,41]. This is in line with the expectation that any adaptive biological process should also be accompanied by trade-offs between robustness and vulnerability [77]. As the old saying goes: “there’s no such thing as a free lunch”, even for a cancer cell. An important requirement to estimate such robustness trade-offs should involve the identification of dynamic features that a cancer signaling system can potentially exploit to gain further robustness. Despite the appeal of these ideas, independent validations *in vitro* and *in vivo* will be crucial to assess the potential clinical value of these hypotheses and theoretical models.

Systems-level weaknesses can also be interpreted as natural byproducts of adaptive, highly robust systems [78]. This in part follows the reasoning that cancer cells take advantage of the same robustness-enhancing mechanisms that normal cells exploit to maintain their physiological states. This can be observed in different signaling systems. For instance, the same feedback loops that normal cells use to protect against hypoxia are the same strategies hijacked by cancer cells to promote their own survival. In hypoxic conditions, cancer cells can overexpress HIF1 (hypoxia-inducible factor 1-alpha), which subsequently results in the up-regulation of VEGF (vascular endothelial growth factor), MMPs (matrix metalloproteases) and chemokine receptors. These activations eventually elicit angiogenesis and cell migration for the benefit of normal and cancer cells alike [79,80].

A reduction of the robustness of the aberrant EGFR-driven signaling network will necessarily require an understanding of its dependence on (or cross-communication with) other RTK signaling systems. In GBM, the prospect of overcoming resistance by inhibiting multiple RTKs has already been reported [43]. The “switching” among signaling systems as a source of resistance may be attributed not only to the re-wiring of the downstream signaling activity of a particular RTK-specific system [81], but also to the expression of multiple RTK ligands [82]. A recent investigation showed that the exposure of patient-derived (lung and breast) cell lines to multiple RTK ligands can induce resistance to TKI treatment [83]. This shows that drug resistance may be established by simply increasing the concentration of one or more ligands, such as HGF (hepatocyte growth factor) and EGF, in a combinatorial fashion. Nevertheless, these types of resistance-inducing mechanisms are by-products, rather than the causes, of the systems-level properties discussed above. It is also important to caution that many instances of “signaling rewiring” may be either causal factors or simple by-products of systems-level robustness. Future research will continue elucidating the specific contexts in which these mechanisms take place.

Several efforts that demonstrate the potential of systems-level targets and therapeutic approaches have been recently reported in glioblastoma. For instance, Zhang et al. [84] described how the combination of nimotuzumab (a monoclonal antibody for EGFR) and an inhibitor of miR-21 reduces tumor proliferation and invasion in comparison to nimotuzumab treatment alone, *in vitro* and *in vivo*. This treatment approach is based on the idea of disrupting a feedback loop connecting miR-21 and EGFR via interaction with PPARα and VHL. Siebzehnrub et al. [85] showed that key regulators of cancer “stemness”, e.g., SOX2, is regulated by a feedback circuit defined by ZEB1 and miR-200. Their research shows the potential clinical value of this regulatory loop by connecting it with invasion, chemoresistance and survival in glioblastoma patients. Although these studies will require further investigations in humans, they are encouraging demonstrations of the power of “regulatory circuits” as candidate therapeutic targets, beyond the traditional “one target – one drug” approach.

Promising examples of systems-directed therapeutic strategies are also available in other cancer research domains, including liposarcoma, breast, bone and lung cancers [38,86-90]. These studies do not only explore and apply systems biology concepts (e.g., data-driven identification of feedback loops and inter-module “crosstalk”), but also take advantage of extensive “multi-omics” network-driven modeling. Despite their usefulness and clinical potential, further validations *in vivo* are required. Also the underlying computational methods may heavily depend on context-specific prior knowledge for model generation, rely on relatively large amounts of signaling measurements and tend to be biased toward particular signaling targets.

The complexity of these challenges demands a solid involvement of advanced computational modeling, from the very early stages of experimental design to subsequent validations. Despite progress achieved in the computational modeling of signaling networks [71,91-94], major hurdles exist to demonstrate its relevance in pre-clinical or translational applications. Among the hundreds of components and interactions specified in comprehensive surveys of this system [95], there are relatively limited amounts of data to estimate network activity and connectivity that are specific to different signaling time scales and sub-cellular localizations. Moreover, models with potential translational applications will require high-resolution tissue-specific quantitative information, which will not be accessible to the vast majority of the research community in the near future. Even if larger amounts of such data become available through advances in high-throughput “omics” technologies, computational researchers will be constrained by the need to implement models that are both biologically meaningful and computationally tractable. Despite these challenges and limitations, computational models will prove useful at least at helping experimental biologists to synthesize published knowledge. Furthermore, they will represent relatively inexpensive predictive tools to explore “perturbation-to-outcome” spaces in a systematic way, which may be more expensive to implement *in vivo* or *in vitro*. More concretely, targeted and combinatorial perturbations performed *in silico* can offer novel clues about resistance in the EGFR and other RTK signaling systems. The simulation of inhibition, knock-down or “rewiring” of signaling components, either permanently or transiently, are examples of the possibilities offered by systems-level computational models.

## Conclusions

Here we have reviewed the EGFR-driven signaling network and important aberrations in GBM. We discussed fundamental properties that can make it resistant to treatment, and highlighted examples of manifestations of these properties in GBM. The ultimate objective is to identify new treatment strategies, which could either impair robustness against stimuli or outsmart compensatory survival mechanisms. In order to outplay this adaptive resilience, researchers will require deeper understandings of signaling responses to perturbations at a systems level.

Potential vulnerabilities in the diseased system may be viewed as by-products of the complexity and robustness that is also inherent in the “normal” signaling system. Moreover, resistance-enhancing properties and mechanisms can be cell-specific and genotype-dependent, and act in close partnership with other RTK and growth-induction signaling systems. As we progress in the elucidation of the systems-level properties that contribute to resistance, an essential step forward will be the further integration of multiple informational views of signaling activity in GBM, before and after treatment. This will not only facilitate advanced levels of mechanistic and quantitative understanding, but also the delineation of new ways to decrease the innate and acquired capacity of tumor cells to resist therapeutically-relevant perturbations.
